# Atmospheric plasma treatment: an alternative of HF etching in lithium disilicate glass-ceramic cementation

**DOI:** 10.3389/fbioe.2023.1259707

**Published:** 2023-12-01

**Authors:** Xiaoqian Wu, Kun Liu, Rui Luo, Jianhao Xu, Mingsheng Chen

**Affiliations:** ^1^ Stomatological Hospital of Chongqing Medical University, Chongqing, China; ^2^ Chengdu Women’s and Children’s Central Hospital, School of Medicine, University of Electronic Science and Technology of China, Chengdu, China; ^3^ The State Key Laboratory of Power Transmission Equipment and System Security and New Technology, Chongqing University, Chongqing, China

**Keywords:** lithium disilicate, non-thermal plasmas, resin cements, shear bond strength, wettability, X-ray photoelectron spectroscopy (XPS)

## Abstract

**Objectives:** This study aimed to investigate whether the atmospheric pressure plasma jet (APPJ) could modify the surface of lithium disilicate glass ceramics (LDC) instead of hydrofluoric acid (HF) in LDC resin cementation.

**Methods:** Two hundred and thirty-two LDC blocks were randomly divided into seven groups: Group 1 (16 specimens) was the blank control group (without HF or APPJ treatment); Group 2 (36 specimens) was etched by HF; Groups 3–7 (36 specimens each) were treated with APPJ, and the relative air humidity (RAH) of the discharge was 22.8%, 43.6%, 59.4%, 75.2%, and 94.0%, respectively. Three LDC blocks in each group were characterized via X-ray photoemission spectroscopy (XPS) analyses, 3 blocks via contact angle measurements, and other 10 blocks via surface roughness measurements. The residual LDC blocks in groups 2–7 were cemented to composite cylinders. Testing the cemented specimens’ shear bond strength (SBS) before and after thermocycling (6,500 cycles of 5°C and 55°C) revealed fracture patterns. Data were analyzed by ANOVA (*post hoc*: Bonferroni) (*α* = 0.05).

**Results:** After APPJ treatment, the water contact angle values of APPJ treated blocks dropped from 31.37° to 5.66°, while that of HF etched ones dropped to 18.33°. The O/C ratio increased after HF etching or APPJ treatment according to the calculated results, except for the APPJ-treated samples at a RAH of 22.8%. The surface roughness of LDC blocks showed no statistic difference before and after APPJ treatment, but experienced significant difference after HF etching. The O/Si and O/C ratios varied after HF etching or APPJ treatment. No significant difference in SBS values could be found among groups 2–7 before or after artificial aging (*p* > 0.05). All specimens showed mixed failure patterns.

**Conclusion:** The APPJ treatment method reported in this study is a promising novel strategy for surface modification of the LDC. With acceptable bonding strength, it might be an alternative to HF in LDC-resin cementation.

## 1 Background

Hydrofluoric acid (HF) is recommended for resin-adhesive cementation of silica-based dental ceramic restorations (Shifra et al., 2021). Lithium disilicate glass-ceramics (LDC) is one of the most extensively used silica-based dental ceramics due to its excellent aesthetics and high biaxial strength (Willard and Gabriel Chu, 2018). The resin-adhesive cementation provides better marginal sealing, reliable retention, and improved fracture resistance and is regarded as the optimal choice for dental ceramic restoration (Braga et al., 1999; Blatz et al., 2003; Ammar et al., 2023). Increasing ceramic surface roughness by HF etching promotes micromechanical interlocking. However, HF is a severe hazard to the human body and the environment (Janda et al., 2003). Common harms of HF include skin and respiration tract burns, systematic fluorosis, and electrolyte imbalance (Wang and Dai, 2019; Mao et al., 2022). Masks, glasses, and acid-proof gloves are used to protect patients and dentists, while sodium bicarbonate is used to neutralize liquids containing HF. HF can also decrease the mechanical strength of the ceramic due to the modification of the resident flaw population (Addison et al., 2007; Luo et al., 2014; Venturini et al., 2015; Venturini et al., 2018), while over-etching (i.e., higher exposure time and acid concentration) can negatively affect the long-term success of ceramic restorations.

Plasmas are partially ionized gases containing electronically excited atoms, molecules, ions, and free radicals. These highly reactive particles can quickly introduce various chemical functional groups on the surface of substrates (Liu et al., 2016). Many types of plasma devices have been developed for industrial applications (Zou et al., 2004; Dong et al., 2005; Khorram et al., 2015). However, their application in medical fields was limited before the invention of the atmospheric pressure plasma jet (APPJ), as conventional plasmas could not be discharged in an open environment and usually showed a very high temperature. Since APPJ can generate plasmas at room condition (Tendero et al., 2006), plasma treatment now has several applications in dentistry, such as teeth whitening (Claiborne et al., 2014; Seoul-Hee et al., 2021), inactivation of bacteria (Hong et al., 2019; Özdemir et al., 2023), treatment of dental caries (Sladek et al., 2004; Sladek et al., 2007), surface modifications of implants (Silva et al., 2011; Canullo et al., 2016; Rutger et al., 2023), and improving dental bonding system (Chen et al., 2012; Chen et al., 2013; Chen et al., 2014).

Scientists have tried to improve ceramic-resin bonding by the APPJ since APPJ surface engineering improves ceramic surface wettability and permeability. Derand et al. (2005) reported that deposition with plasmas on the zirconia ceramic significantly increased the bond strength of ceramic-resin cementation. Cho et al. (2011), Han et al. (2012) treated a feldspathic ceramic with an APPJ after triethyleneglycoldimethacrylate coating, increasing the hydrophilicity of the treated ceramic surfaces and contributing to adhesion. The bonding of LDC to resin might also be improved by APPJ treatment, as observed with glass (Abenojar et al., 2013). In this study, an Argon APPJ was employed, which operated under room temperature, and the discharge’s relative air humidity (RAH) could be altered. The null hypothesis is that APPJ, compared with HF, could not improve the bonding strength between LDC and resin in cementation.

## 2 Methods

### 2.1 Specimen Preparation

Two hundred and thirty-two LDC blocks (8 mm × 8 mm × 4 mm, IPS e.max^®^ Press, Ivoclar Vivadent, Schaan, Liechtenstein) and 120 composite resin cylinders (3.5 mm in diameter and 3 mm in thickness, Filtek Z350, 3M ESPE, United States) were fabricated according to the manufacturer’s recommendations. The LDC blocks were polished with a polishing machine (Exakt 400 CS, Exakt Apparatbau, Norderstedt, Germany) using SiC sandpaper (991A, Matador, Germany) in the sequence of 200, 400, 600, and 800 grit. Subsequently, the LDC blocks were ultrasonically cleaned (KQ-500DE, Kunshan Ultrasonic Instruments Co., LTD., Jiangsu, China) for 5 min with absolute ethanol (Analytical Purity), air-dried, and then stored in dry plastic containers. The composite cylinders were polished with sandpaper of 200 and 400 grit accordingly, cleaned, and stored in the same way as the LDC blocks.

### 2.2 LDC blocks treatment

**FIGURE 1:**
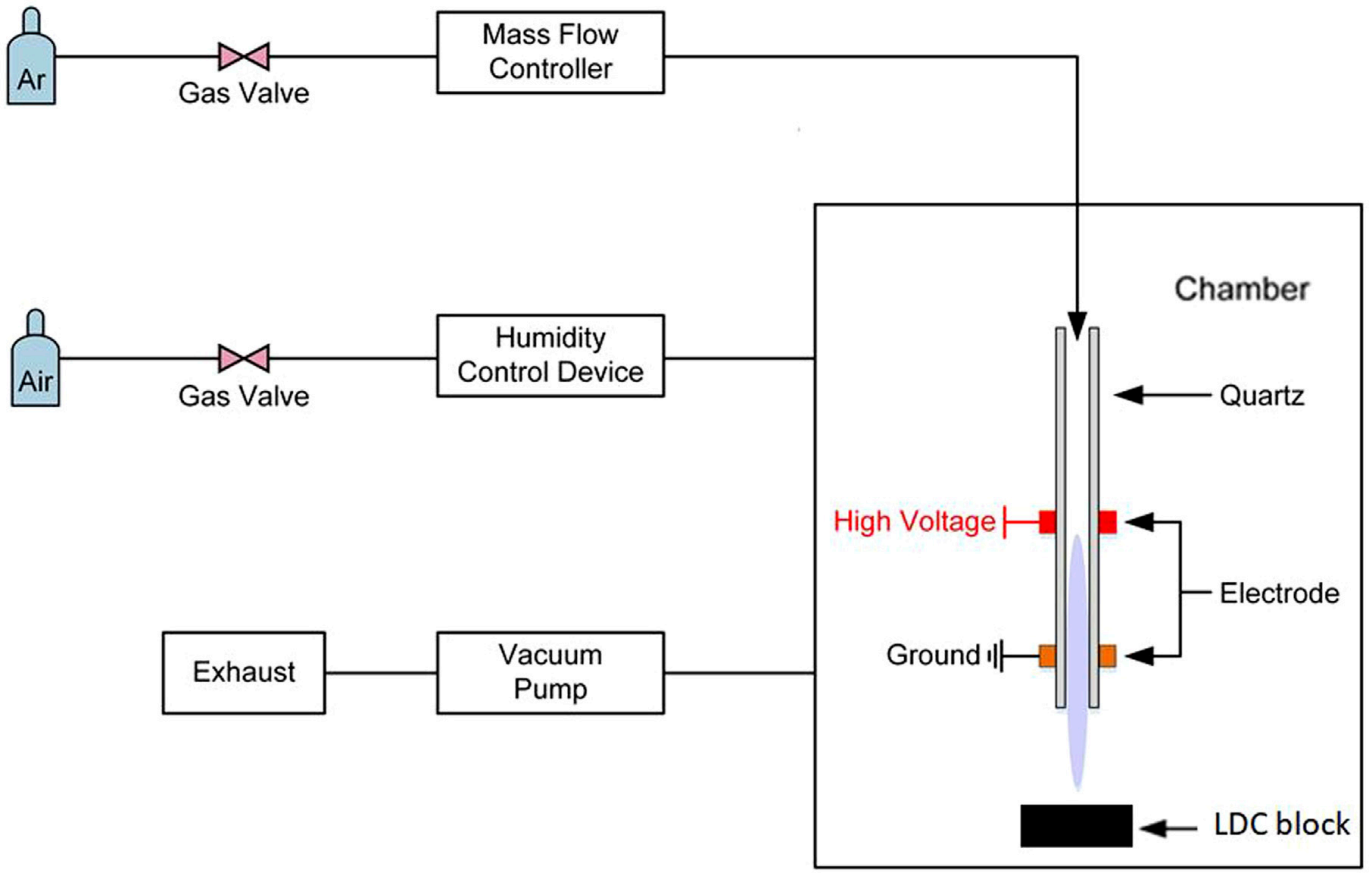
Schematic diagram of the atmospheric pressure plasma jet system.

This study employed an APPJ developed by Liu (Liu et al., 2019). The jet is an atmospheric pressure dielectric barrier discharge argon plasma instrument, driven by an AC high voltage power supply at a discharge voltage of *V*_*pp*_ *=* 26 kV, a frequency of *f =* 9.6 kHz, an argon flow rate of three standard liters per minute, as shown in Figure 1. An air-tight chamber and the humidity control device altered the RAH of the discharge, which was used to simulate the RAH in a wide range in different application scenario. They would be abandoned in real clinical use. A mobile base allows the operator to keep a distance of 10 mm between the nozzle and LDC surfaces.

Two hundred and thirty-two LDC blocks were randomly divided into seven groups. Group 1 (16 specimens) was the blank control group (without HF-etched or APPJ-treated). Group 2 (36 specimens) was etched with HF. Groups 3–7 (36 specimens each) were treated with APPJ. The blocks in group 2 were etched with 9.5% HF gel (Bisco Inc., Schaumburg, IL, United States) for 20s, rinsed thoroughly with tap water for 1 min, ultrasonically cleaned in absolute ethanol for 5 min, and air dried at room temperature—following manufacturer’s instructions. The blocks in groups 3–7 were subjected to the APPJ processes for 30s per specimen with different RAH, which were 22.8%, 43.6%, 59.4%, 75.2%, and 94.0%, respectively. Three LDC blocks in each group were characterized via X-ray photoemission spectroscopy (XPS) analyses, 3 blocks via contact angle measurements, and other 10 blocks via surface roughness measurements.

### 2.3 Bonding procedure

After HF etching or APPJ treatment, the LDC blocks were bonded to composite cylinders immediately using a resin bonding system (BISCO Inc., United States). First of all, a very thin adhesive tape with a perforation (*ϕ* = 3.7 mm) in the center was applied on the treated LDC surface, so that the bonding area would be confirmed. Parts A and B of Bis-Silane were added to the mixing well, stirred, brushed onto each bondable LDC surface, waited for 30s, and dried with warm air. Next, a thin layer of Porcelain Bonding resin was applied to these LDC surfaces. This was followed by applying a dual-cured resin luting cement to each bondable LDC surface. Next, the LDC was bonded to the corresponding composite cylinder. The specimens were pressurized under a 10 N load for 30s, excess resin cement was carefully removed, and the specimens were polymerized (1,200 mW/cm^−2^, Ivoclar-Vivadent AG, Schaan, Liechtenstein) for 40s, according to the manufacturer’s instructions.

### 2.4 Shear bond strength (SBS) test and fracture analysis

All 120 cemented specimens were stored in distilled water at 37°C in an incubator (Forma 3111, Thermo Fisher Scientific, United States). Forty eight hours later, 10 specimens in each group were subjected to an SBS test. The remaining 10 specimens were subjected to an SBS test after 6,500 thermocycles at 5°C and 55°C with a 30-s dwell time and a 2-s transferring time for each distilled water bath [TC 501F(III), Weier Inc., Suzhou, China]. A universal testing machine (C43.104, MTS, United States) was used for the SBS test at a 0.5 mm/min crosshead speed. The maximum load of each specimen was recorded. The SBS was calculated as follows:

SBS (MPa) = fracture load/bonding area.

The fracture patterns were analyzed optically using a stereoscopic microscope (SteREO Discovery V20, Zeiss, Germany). The following were assigned as debonded surfaces: a) cohesive failure (the fracture occurred within the resin cement), 2) adhesive failure (the resin cement was completely removed), 3) mixed failure (both fracture patterns could be observed).

### 2.5 Surface contact angle measurement

The wettability of the LDC surfaces to water was determined with static contact angle measurements. After different treatments, three specimens in each group were tested with a contact angle goniometer (SCI3000F, Huanqiu Hengda Technology Co., Ltd., China). A syringe tip was placed above the specimen stage, and a drop of 1 μL ultra-pure water was dispensed on each tested surface. A photo was taken 5 s later as the droplet arc became stable. The angles of contact were traced and recorded to be analyzed.

### 2.6 XPS measurement

After different treatments, three specimens from each group were examined with XPS (ESCALAB 250Xi, Thermo Fisher Scientific, United States), using monochromatized A1Kαradiation (1486.6 eV photon energy, energy step size 0.05 eV). Each specimen was analyzed two replicates, and the value was averaged to obtain the reported atomic percent (at%). The data were analyzed with Thermo Scientific Avantage 5.976 software (Thermo Fisher Scientific, United States).

### 2.7 Surface roughness measurement

After different treatments, 10 specimens from each group were examined with an ultra precision measurement system (Form Talysurf, PGI 1200, Taylor Hobson Precision, United Kingdom). Calibration and leveling were carried out before the measurement. A jig was applied to make the blocks in position. Each specimen was examined separately in perpendicular directions, and the average value was recorded.

### 2.8 Statistical analysis

Shapiro-Wilk test was used to test the normality of data distribution. The SBS values, the contact angles and surface roughness values were analyzed using the One-way or Two-way analysis of variance (ANOVA) followed by Bonferroni *post hoc* tests (SPSS Version 25.0, Chicago, IL, United States). All tests were performed with a significance level of *ɑ* = 0.05.

## 3 Results

### 3.1 SBS test and failure pattern

**FIGURE 2:**
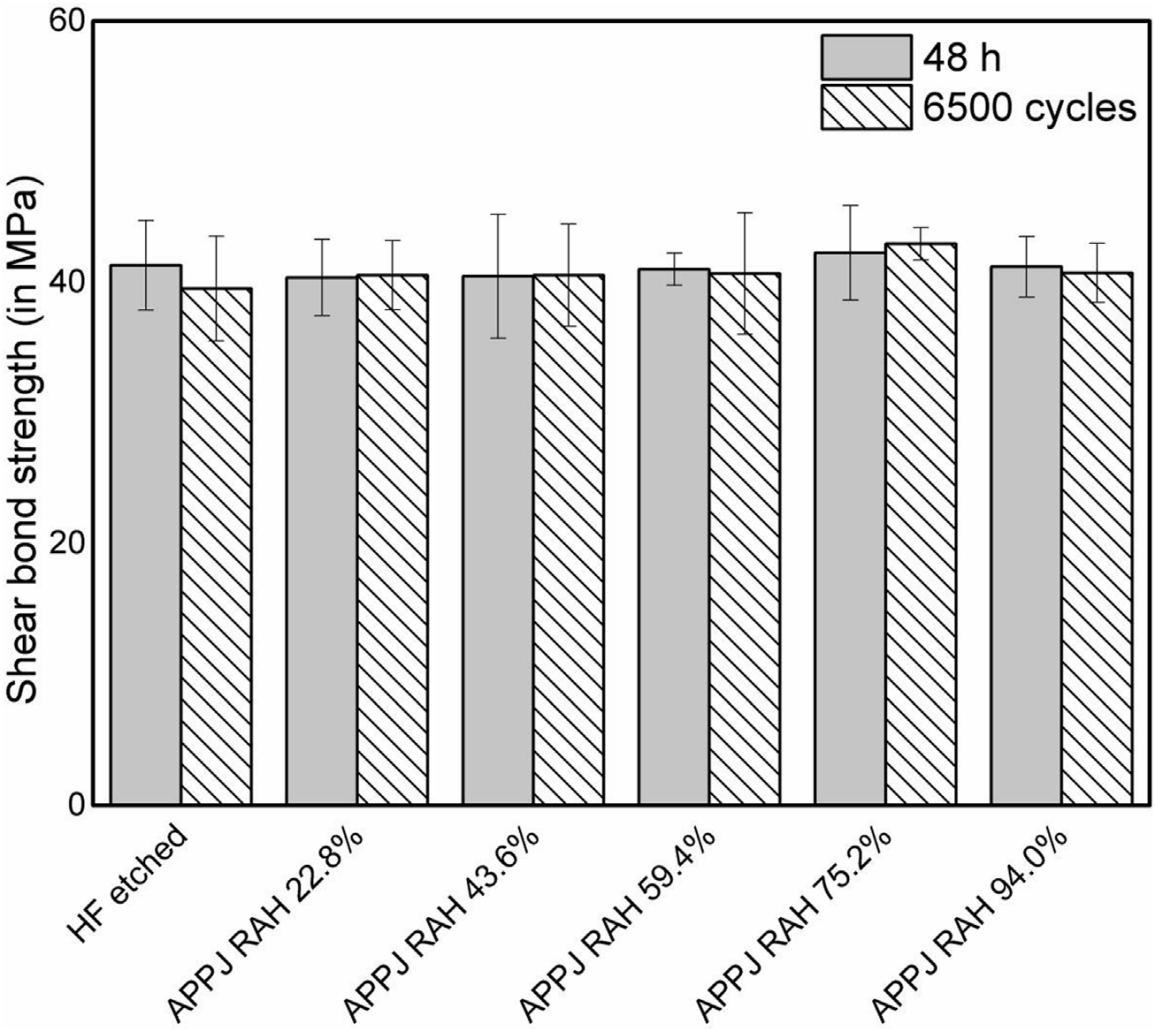
Shear bond strength (in MPa) of resin composite to LDC surfaces in 48 h evaluations and after 6,500 thermocycles. Bars represent mean while whiskers represent standard deviation.

**FIGURE 3:**
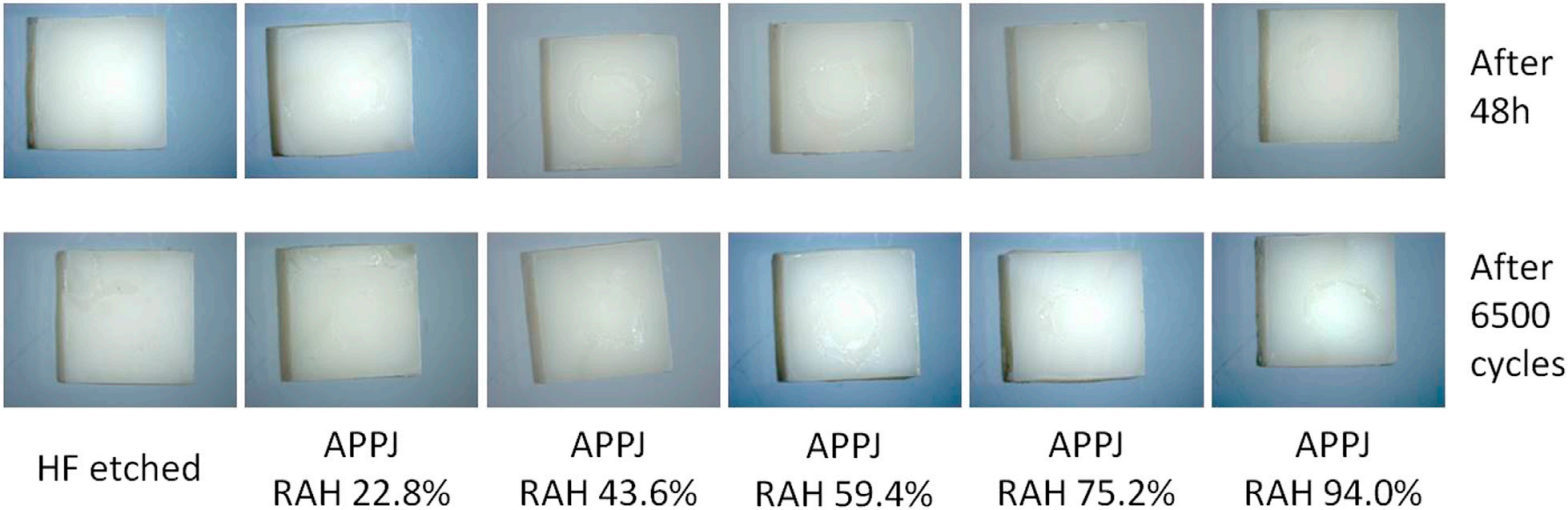
Representative images of fractured surfaces.

The results of the SBS measurements were presented as bar charts with standard deviations in Figure 2. After 6,500 thermocycles, the SBS of the HF-etched group decreased slightly from 41.31 to 39.53 MPa. The SBS of APPJ-treated groups varied with RAH: 40.37–40.55 MPa at a RAH of 22.8%; from 40.47 to 40.55 MPa at a RAH of 43.6%; from 41.00 to 40.68 MPa at a RAH of 59.4%; from 42.26 to 42.95 MPa at a RAH of 75.2% (these were the highest values of both before and after thermocycling process); and from 41.19 to 40.73 MPa at a RAH of 94.0%. Two-way ANOVA (*post hoc*: Beniferroni) revealed no statistical difference in the SBS of all groups, regardless of the thermocycling process or the treatment (*p* > 0.05). All specimens presented mixed failures before and after 6,500 thermocycles (Figure 3).

### 3.2 Surface contact angle measurement

The water contact angle on LDC surfaces in different groups is shown in Table 1. The contact angle of water was 31.37° ± 1.57° on the surfaces of blank control group, and it was 18.33° ± 1.57° on the surfaces of HF-etched group. After APPJ treatment, the water contact angle values dropped dramatically. At 22.8% RAH, the contact angles decreased to 23.69° ± 2.01°. The values reached the minimum of 5.66° ± 1.93° and then increased slightly to 7.36° ± 1.54° at 75.2% and 94.0% RAH, respectively. No statistical difference was observed among the APPJ-treated groups at RAH of 59.4%, 75.2%, and 94.0% (*p* > 0.05).

**TABLE 1:** Values of water contact angle of LDC surfaces.

Treatment	Water contact angle (°)
Blank control (no treatment)	31.37 (1.71)^a^
HF-etching	18.33 (1.57)^b^
APPJ treatment-RAH 22.8%	23.69 (2.01)^c^
APPJ treatment-RAH 43.6%	19.26 (0.82)^b^
APPJ treatment-RAH 59.4%	6.37 (0.90)^d^
APPJ treatment-RAH 75.2%	5.66 (1.93)^d^
APPJ treatment-RAH 94.0%	7.36 (1.54)^d^

Values are given as mean (SD).

Mean values with the same uppercase superscript letters indicate no significant difference.

### 3.3 XPS measurements

**FIGURE 4:**
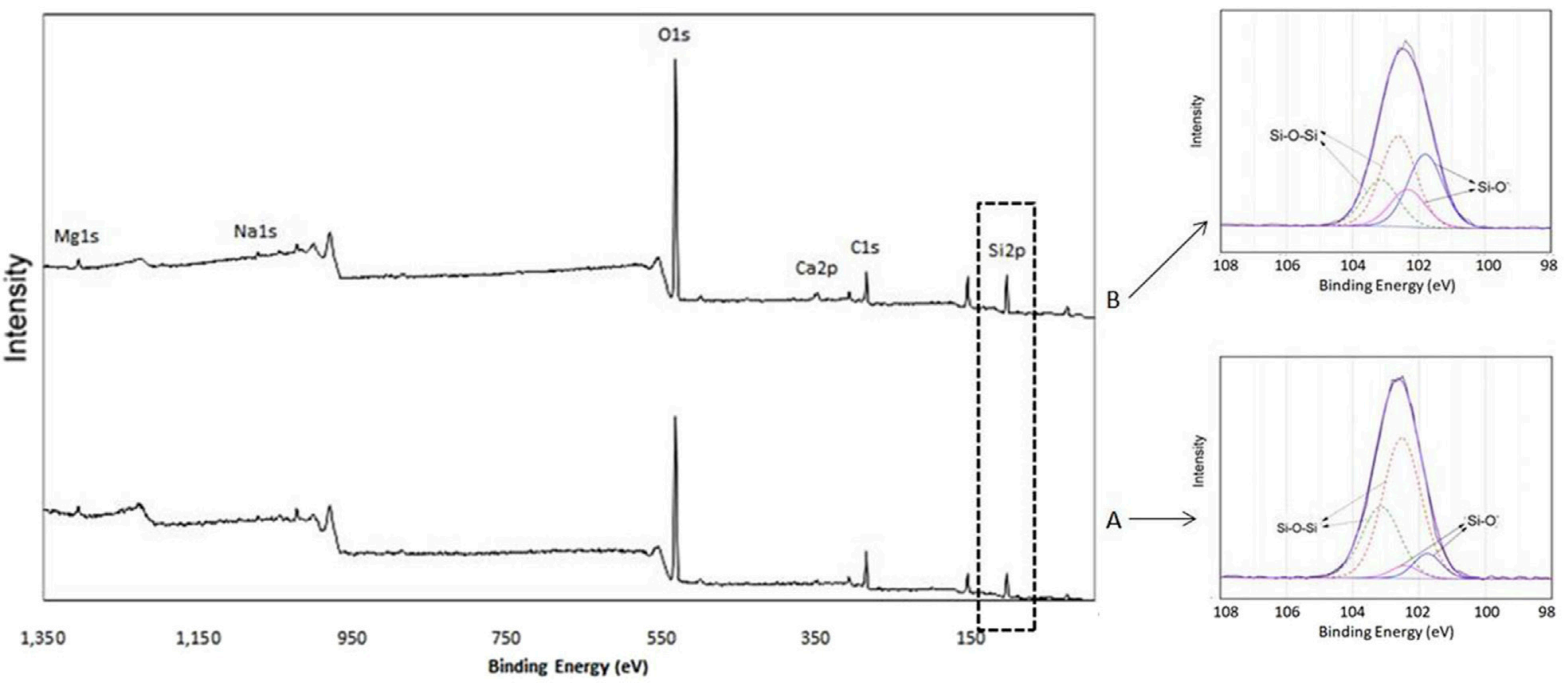
The high-resolution spectra of Si2p3/2. **(A)** Untreated sample (group 1, blank control); **(B)** APPJ-treated sample (group 6, RAH = 75.2%).

XPS measurements were performed to analyze the atomic compositional changes of the LDC surfaces. Quantitative atomic percent concentrations for all seven groups are shown in Table 2. The representative XPS spectra of the blank control group revealed that most dominant peaks were associated with the O 1s and C 1s located at binding energies of ∼533 eV peak and ∼285 eV, respectively. The atomic percent of carbon (at %C) of the untreated LDC surfaces was about 40.90%. The strongest carbon reduction on the surface was achieved by HF etching, decreasing the at %C to 12.57% from 40.9%. The atomic percent of oxygen (at %O) of untreated LDC surfaces was about 41.11%, which increased to 61.33% after HF etching. The detailed at %C and at %O of APPJ-treated samples are shown in Table 2. Other elements, such as Si, Ca, and Zn, were also detected. The O/C ratio increased after HF etching or APPJ treatment according to the calculated results, except for the APPJ-treated samples at a RAH of 22.8%. There is no obvious signal for Li, as Li_1s_ peak has very low sensitivity. Large number (50) of scans should be used when acquiring Li_1s_ spectrum, however, there was only a few scans in this measurement. The high-resolution spectra of Si is shown in Figure 4.

**TABLE 2:** Surface chemical composition (in at% of each element) of LDC surfaces.

Elements	O_1s_	C_1s_	Si_2p_	Na_1s_	Ca_2p_	Zn_2p_	O/C	O/Si
Blank control (no treatment)	41.11 (5.37)	40.90 (5.79)	16.61 (0.40)	ND	0.30 (0)	1.08 (0.22)	1.01	2.48
HF etched	61.33 (0.14)	12.57 (0.36)	25.00 (0.32)	1.00 (0.46)	ND	ND	4.88	2.45
APPJ—RAH 22.8%	41.01 (3.36)	41.13 (5.28)	16.30 (1.89)	ND	0.89 (0.08)	0.67 (0.07)	1.00	2.52
APPJ—RAH 43.6%	54.57 (0.49)	24.34 (0.57)	20.55 (0.08)	ND	ND	0.54 (0)	2.24	2.66
APPJ—RAH 59.4%	52.62 (2.16)	25.81 (3.67)	19.18 (1.39)	0.58 (0.03)	1.24 (0.14)	0.57 (0.11)	2.04	2.74
APPJ—RAH 75.2%	55.49 (6.08)	21.42 (8.27)	20.51 (2.19)	0.75 (0.16)	1.26 (0.19)	0.57 (0.05)	2.59	2.71
APPJ—RAH 94.0%	52.93 (0.94)	24.16 (1.51)	20.52 (0.58)	0.77 (0.16)	0.69 (0.32)	0.93 (0)	2.19	2.58

ND: not detected.

Values are given as mean (SD).

### 3.4 Surface roughness measurement

Surface roughness measurement was carried out to reveal the effect of different treatments on the LDC surfaces. The surface roughness of each group is shown in Table 3. The value of blank control group was 0.1943 (0.0614) μm, and group 3–7 (APPJ treated ones) showed no significant difference. But when it comes to group 2 (HF etching), the value increased to 0.2854 (0.0253) μm.

**TABLE 3:** Surface roughness of LDC sufaces

Treatment	Surface roughness (μm)
Blank control (no treatment)	0.1953 (0.0614)^a^
HF-etching	0.2854 (0.0253)^b^
APPJ—RAH 22.8%	0.2018 (0.0422)^a^
APPJ—RAH 43.6%	0.2015 (0.0442)^a^
APPJ—RAH 59.4%	0.2120 (0.0657)^a^
APPJ—RAH 75.2%	0.1961 (0.0308)^a^
APPJ—RAH 94.0%	0.1851 (0.0342)^a^

Values are given as mean (SD).

Mean values with the same uppercase superscript letters indicate no significant difference.

## 4 Discussion

This study investigated the effects of APPJ on the SBS (before and after artificial aging), surface wettability, and surface elemental composition of LDC. No significant difference in SBS values could be found among APPJ-treated and HF-etched groups, neither before nor after artificial aging by thermocycling. The failure modes of debonded specimens in all groups showed mixed (adhesive and cohesive) failure patterns, indicating that APPJ treatment improved the SBS stably, just like the classic HF etching. The average SBS values varied slightly with the change in RAH, reaching the highest point at a RAH of 75.2%. No significant difference was observed among the APPJ-treated groups before or after artificial aging, indicating that the RAH of the environment had no significant effect on the SBS. These results rejected the null hypothesis. As it is generally assumed that a shear bond strength at 18–20 MPa is acceptable for the clinical requirements of adhesive dentistry (Hannig et al., 2006), the SBS results of APPJ treatment groups is acceptable.

**FIGURE 5:**
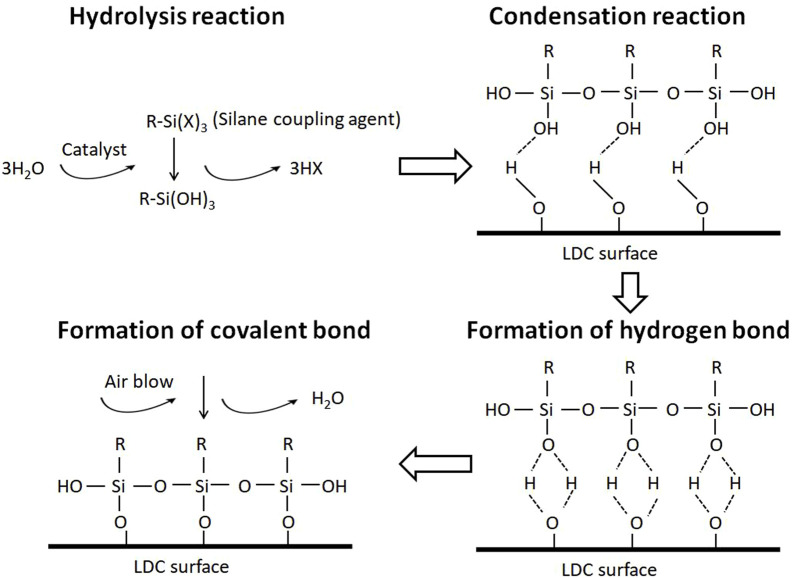
Schematic diagram of the reaction between LDC and silane coupling agent.

The bonding strength of LDC to resin can be improved in two ways: 1) Increasing the bonding area through HF etching. However, there are many problems in HF applications (Janda et al., 2003; Addison et al., 2007; Luo et al., 2014; Venturini et al., 2015; Venturini et al., 2018). 2) Changing the surface chemical property by applying silane coupling agents, as shown in Figure 5. Silane coupling agents are organosilicon compounds that can produce active groups under specific conditions. The activated silane strengthens the interface by two synergistic bonding mechanisms: increasing the wettability of the LDC surface and chemical adhesion (Lung and Matinlinna, 2012).

A water contact angle test was performed to evaluate the wettability of the LDC surfaces. When the samples were subjected to APPJ treatment, the water contact angle on the LDC surfaces was significantly reduced than the HF-etched samples, suggesting that the surface of LDC became more hydrophilic in a short time. This result was consistent with that reported for dental substrates and glass (Abenojar et al., 2013; Liu et al., 2016). The contact angle of group 6 decreased to the minimum value of 5.66 ± 1.93 at a RAH of 75.2%, while that of the untreated and the HF-etched group were 31.37 ± 1.71 and 18.33 + 1.57°, respectively. Further, an increase in group 7 (RAH 94.0%) led to a slightly weakened effect, as reflected in a slight increase in contact angle (7.36° ± 1.54°). This change was consistent with the shift in the SBS values, which changed positively with the variation of the surface wettability in the APPJ-treated groups. However, there was no significant difference in SBS values among all treated groups (*p* > 0.05).

XPS was employed to determine the chemical composition of the LDC surfaces. No Ar signal could be found in all APPJ-treated groups, indicating that argon was not integrated into the LDC surfaces. The results revealed an increase in the O/C ratio after HF etching or APPJ treatment, consistent with previous studies (Abenojar et al., 2013; Chen et al., 2013; Valverde et al., 2013; Lee et al., 2017). For instance, the O/C ratio increased from 1.01 to 2.59 in group 6 (at RAH of 75.2%). It is well known that APPJ is rich in reactive oxygen species such as •OH and O. It promotes the incorporation of functional oxygen-containing groups (C-O, C-OH) into the upper layer of the treated yttrium-stabilized zirconia surface (Silva et al., 2011). Since the substrate is more active, more hydroxyl groups might integrate into the APPJ-treated LDC surface. When a silane coupling agent was applied, these groups might react with it, leading to more chemical bonding between the LDC and resin cement.

LDC contains silicate tetrahedron chains with some oxygen atoms acting as bridges between silicon atoms (Si-O-Si) while others do not (Si-O-). The O/Si ratio in Si-O- and Si-O-Si bonds was 1:1 and 2:1, respectively (Abenojar et al., 2010). Figure 4 shows the Si_2p_ high-resolution spectra of untreated (group 1, blank control) and APPJ-treated (group 6, RAH = 75.2%) samples. The Si_2p_ peak centered at 102.6 eV was deconvoluted using Gaussian-Lorenztian into four peaks: two each from Si_2p3/2_ and Si_2p1/2_ with an energy difference of 1 eV. The Si_2p3/2_ component (at 101.8 eV) was assigned to Si-O- bond (silicon bonded with non-bridging oxygen), and the Si2_2p3/2_ component at (102.8 eV) to the Si-O-Si bond (silicon bonded with bridging oxygen), respectively (Abenojar et al., 2010; Abenojar et al., 2013). The deconvoluted spectra after APPJ treatment show an increase in the percentage of Si-O- bonds due to the increase in Si-O-H, providing more hydroxyl groups for the chemical bonding between LDC and silane.

Surface roughness is also a very important factor on the bonding strength. A rough surface would provide more bonding area microscopically, thus expands the micro-interlock between ceramics and resin, which eventually manifested as the increase in bonding strength (Romanini-Junior Jose et al., 2018). The surface roughness measurement showed that the APPJ treatment, no matter at any relative air humidity, would not alter the surface topography details. This result is similar to our previous research on dental zirconia (Liu et al., 2019). As dental prosthesis becomes more and more accurate, the dentists would prefer to keep the accuracy in their clinical practice. However, HF etching would change the LDC surface a lot, while HF etching procedure is so technically sensitive that insufficient or excessive etching would be harmful to the bonding (Addison et al., 2007; Venturini et al., 2015). The HF etched surface showed a much higher roughness value than other groups in this experiment (Table 3).Therefore, the surface roughness measurement suggested that the increase in SBS after APPJ treatment is not mainly contributed by the micro-interlock between adhesive and ceramic, while micro-interlock contributes a lot in the HF etched ones. The story is similar in the influence of different treatment to water contact angle values (Table 1).

Among the conditions affecting plasma generation, temperature and pressure directly influence plasma generation’s feasibility in a dental clinic (Ito et al., 2016). In this study, the plasmas are generated from the gas at atmospheric pressure and not very high temperature. The relative air humidity would not influence the shear bond strength of LDC cementation. Further, dentists or patients do not directly come in contact with the plasmas, as it is operated *in vitro*. This plasma generator is small and light in weight and can be hand-held and operated by dental practitioners in clinics.

## 5 Conclusion

Within the limitations of this study, it was concluded that the APPJ treatment was effective for surface modification of the LDC to obtain acceptable bonding strength and might be an alternative to the HF etching.

## Data Availability

The original contributions presented in the study are included in the article/Supplementary Material, further inquiries can be directed to the corresponding author.

## Abbreviations

APPJ, atmospheric pressure plasma jet; LDC, lithium disilicate glass-ceramics; HF, hydrofluoric acid; RAH, relative air humidity; SBS, shear bond strength; XPS, X-ray photoemission spectroscopy.
